# The Air We Breathe1Read before the Buffalo Medical Union, October 17, 1906.

**Published:** 1907-01

**Authors:** De Witt C. Greene

**Affiliations:** Buffalo, N. Y.


### The Air We Breathe.[1]

By DE WITT C. GREENE, M. D., Buffalo, N. Y.

IN taking up the subject of the air we breathe, there are several points to consider, most prominent of which are what is pure air, and what renders air impure? Among the causes of impurity we have vitiation by passing through the lungs, by combustion, by effluvia, and vitiation by organic matter, and the like. We will also note some of the effects and remedial measures.

All animal life requires air, that it may thrive and flourish, consequently we are very much at the mercy of our surrounding atmosphere; be it good or bad, we must indulge. It is different with food or drink. If we prefer this or that food or drink it is at our discretion to choose. Not so with the air about us; we must breathe what is furnished at the particular moment.

Air that passes over water in motion and over mountainous localities having foliage is usually the most pure, containing as it does oxygen about 20%, nitrogen 79%, carbonic acid, (ozone, which is not found in cities,) watery vapor variable, salts and organic matter traces. As I just remarked, ozone is not found to any extent in cities, this being the element that gives the air of the country its freshness. Twenty times in a minute each person consumes about one cubic foot of air. Consequently, if people are in a building, ventilation is required according to the dimensions of the room, and number of people present or they will be detrimentally affected. I beleve that more people die from the impurities they breathe than from all other sources combined. When we consider diphtheria, scarlet fever, pulmonary tuberculosis, and similar diseases, how rapidly the mortality runs up. Most of our cities and villages have taken steps to provide ventilation among other requisites for the wholesome home, and today buildings are erected only after the plans have been approved by certain town or city officials, having in mind safety of construction, proper fire protection, and ventilation.

Special attention, too, is being given to the tenement house. Our tenement houses are largely occupied by Italians and Poles, especially in this city, and they are disposed to group themselves in small rooms to reduce the rent, and also because that was their custom in their native land. In Italy, especially about Naples, there may be seen the tenement locality so crowded that one would think the inhabitants from other parts of the city had all congregated in one particular place. There the climate is so mild that ventilation is much better, the windows being open the greater part of the year.

Air in passing through the lungs gives up about 4% of oxygen and about the same amount of carbonic acid is exhaled,

1. Read before the Buffalo Medical Union, October 17, 1906.

besides watery vapor and organic matter; the skin also adding its contribution to the pollution. In twenty-four hours it is estimated that from the respirations alone there are thrown off about one quart of watery vapor, and organic matter. How often when called at night to see a person suddenly taken ill, we find the room of the patient so polluted by vitiated air that we are obliged to throw open a window to get a satisfactory amount of oxygen.

The air of rooms is also often vitiated by the combustion of illuminating gas, and gas used in stoves without chimney connection, so much used in many cities and villages. Gas is perhaps used more in cities and villages for lighting than any other article. It is estimated that the vitiation of air from the effects of one gas jet equals the amount of vitiation caused by three or four persons.

We also have sometimes present in the atmosphere of rooms carbon monoxide formed by the partial combustion of carbon, commonly called coal gas. This is so very poisonous, that 1% is said to have proved fatal. It is remarkable what poor judgment is often exhibited by those in charge of ventilating public places, such as churches, theaters, cars, schools, houses, and other buildings. Often no discretion whatever is used, but either all windows or ventilators are opened or all of them are closed, those in charge not realizing at all that a constant change according to conditions is desirable. Recently, when in one of our public schools I was seated near a radiator. As the room was quite warm I endeavored to turn off the heat. I asked the teacher in charge of the room if I was turning it the right way, and her reply was, that she did not know as she never touched it. Pointing to the registers in the wall, I enquired if the fresh air came in above or below, and to this question she looked blank; and all this after teaching about thirty pupils in this room for at least two years to my knowledge.

The aim of ventilation is to remove the products of combustion and substitute therefor fresh air without draughts. One person requires about 2500 cubic feet of air per hour. A room that is 15 x 16 and 10 feet high contains 2400 cubic feet of air, When one person is in this room, the air to be kept as pure as before he entered it, should be changed every hour. School rooms are seldom provided with adequate means of ventilation and this lack often accounts for dulness and sleepiness of pupils, by reason of taking into their systems vitiated and poisonous air. As a rule the teachers are affected more than the pupils, as the latter, especially if young, run about and throw off the accumulated poisons, while the teacher drags herself to the nearest street car, to be carried home and lies down exhausted from the efforts of the days' work and the vitiated air still in her system.

Sometime ago Dr. Carpenter and Professor Hill of this city made many examinations of the atmosphere of school rooms in this as well as other cities, with the idea of improving the system of ventilation of the schools of Buffalo. In making their observations in the schools they noted the conditions of every room occupied, and then noted the highest and lowest amount of carbonic acid per 10,000 cubic feet of air in each room. Over eight parts of carbonic acid in 10,000 is very undesirable. These are among the observations they made, giving style of ventilation, with lowest and highest percentage of carbonic acid in each room in 10,000 parts.

CARBONIC ACID.

| | | | In 10,000 Parts. | Lowest. | Highest. |
|---|---|---|---|---|---|
| 1st | School | Fan Vent | per vol | 4.62 | 7.82 |
| 2d | " | " | " | 4.05 | 7.7 |
| 3d | " | Window | " | 9.1 | 20.9 |
| 4th | " | " | " | 5.5 | 9. |
| 5th | " | Fans | " | 4 9 | 16. |
| 6th | " | " | " | 6.4 | 10.4 |
| 7th | " | " | " | 4.5 | 14.6 |
| 8th | " | Windows | " | 38. | 52.4 |
| 10th | " | Vacuum | " | 8. | 12.6 |
| 11th | " | Windows | " | 13. | 25.6 |

As only the schools out of town which were supposed to have the best ventilation were examined it would hardly be fair to compare them with the Buffalo schools. I believe they found the Roger Walcott school in Boston, Mass., to average 4.5, it being the best ventilated school of any visited. No wonder with a report of this kind that scholars, boys, as well as girls, often find much difficulty in completing the school year, with the large amount of work exacted of them. Close confinement for long hours in crowded and poorly ventilated school rooms, may produce nervous susceptibility, chlorosis, spread of infectious diseases, and other troubles which could be largely avoided, if fewer scholars occupied a given space and the ventilating apparatus were not overtaxed.

Sadly enough a member of this society lost a son from the effects of pulmonary tuberculosis contracted from a scholar at school who sat in front of him. Carbonic acid, being warmer than the surrounding air, when exhaled rises immediately, and if not allowed to escape cools and settles down only to be breathed again, if not drawn or forced out by mechanical device. Nor is carbonic acid the worst feature of vitiated air, but the putrifying organic matter given off in the breath and by the skin is even more poisonous. We are apt to measure the impurities, however by the amaunt of carbonic acid which the air contains.

There are three mechanical methods of ventilating rooms—namely, vacuum, air forced in at ceiling and withdrawn at floor, and the reverse. In France, the inlet is usually near the floor and outlet near the ceiling. In this country we are inclined to take the opposite method of forcing in the air near the ceiling and drawing it off at the floor. Then we have the vacuum plan of drawing off air from the floor, the fresh air rushing in to take its place. This method is becoming quite popular, and is frequently used in ventilating modern steamships.

Another cause of vitiation of the air we breathe is effluvia. Our cities have established a complete network of leaky sewers. Sewer air is made up of ordinary atmospheric air, gases from fermentation and petrifaction, and a variable amount of organic matter in suspension. The dangers are not so much from gases as from the microorganisms held in suspension. We also have to breathe the contaminated air containing dust. In cities especially the air contains many impurities from decaying material of all kinds and colonies of bacteria. The automobile is a new factor in causing dust; it is not only detrimental to health, but much complaint is made along country roads of its being destructive to vegetation. The leaves of trees and plants, cease to thrive when covered with dust, as they are the lungs of vegetation.

Again, in some cities, and I am sorry to say Buffalo is among the number, we have to breathe in an atmosphere of smoke, but the amount of smoke respired is not always apparent. We cannot fail, however, to always recognize it when we approach the city from any direction, but after being in town for a few days we become somewhat accustomed to it. If a cloud of it drops down on us from some downtown smokestack our attention for a moment is attracted, only to be soon forgotten, and then we go on breathing it just as before. The smoke problem is a difficult one to deal with and it is not new. About six hundred years ago when London had a population of only 50,000 the citizens petitioned King Edward I to prohibit the use of soft coal (which is the kind that today makes the smoke for our city), and he responded by making the use of soft coal an offense punishable by death. We think this a rather severe penalty, but surely the punishment should fit the crime. When we consider that in one ton of coal there are about sixteen pounds of sulphur and this escapes into the air as sulphurous acid, a portion combining with oxygen of the air and forming sulphuric acid there is ample reason for prohibiting the use of soft coal. This acid is always destructive to vegetable and living tissue. The air that is charged with smoke is very injurious, causing as it does many chest affections; besides it is detrimental to foliage and mars the appearance of our monuments and fine buildings.

A boiler user has no right to inflict on a community an unnecessary amount of smoke and gases, simply to save money by using a particular kind of fuel, which is a menace to cleanliness, comfort and health. The man behind the shovel (if the automatic stoker be not used) can do much to relieve the smoke nuisance even when using soft coal. For example, in my neighborhood there is a manufacturing plant which has been in existence for years, during which no annoyance was experienced by neighbors on account of smoke. This plant changed hands May 1, 1906, the new firm continuing to use the same bituminous coal, boiler and engine. Soon the amount of smoke emitted became so great at times that neighbors were obliged to keep their windows closed and send out their laundry. This condition was relieved only after applying to the authorities. It requires an expert to be a good fireman, but the automatic stoker is the more reliable. Anthracite coal is dearer than bituminous, and always will be in this country, as it is found only in Pennsylvania, while bituminous is found in many states. In Pittsburg the smoky conditions have improved only after years of persistent warring on the nuisance. I was told by a carpenter, who some time ago was engaged in the construction of a hotel at Pittsburg, that it was so smoky that each carpenter was given six candles a day in order that he might have light enough to perform his work. Some claim that carbon is healthful. We do not think it improves the healthy lungs, but on the contrary irritates them, and we do know that the gases that accompany the carbon are detrimental to health, as the soot is not simple carbon but is laden with carbonic oxide, carbonated hydrogen, and sulphuretted hydrogen.

In England, firemen and engineers are liable to a fine if their engines smoke unnecessarily, which does much toward preventing the nuisance. Munich has the reputation of being the most free from smoke of any large manufacturing city in the world. Smoke consumers are used there which devour the smoke. In this city among the large plants we find foundries for the manufacture of bells, bronzes, iron, machinery, steel wares, and the like, and about fifty breweries all using much coal. The argument that the presence of smoke indicates business is refuted in the example of Munich which in 1871 had a population of 170,000 and in 1905 of over 500,000.

In Boston there is a law that no boiler will be permitted unless the owner agrees to use hard coal, or a smoke preventer. In Cleveland much trouble was experienced with smoke from railway locomotives, and accordingly a method was employed to keep a record of five different roads running into the city. A chart was used ranging from 0, no smoke, to 100, very dense smoke, and giving the length of time of observation; and by judicious management instead of careless firing, the amount of smoke was reduced from 20% to 11%. This only goes to show what care will do.

In Buffalo, especially in the business section, it is very sooty at times and it is getting worse as time goes on, and this, regardless of the fact that our city ordinance prescribes, "That it is unlawful for any person or persons to maintain or cause to be maintained any engine sending forth a large amount of smoke, causing discomfort and being injurious to health and property." New York city would not tolerate for one moment what we do. The New York Central and Hudson River R. R. running into the city is compelled to use anthracite coal and very little if any bituminous coal is used in the city.

It requires an expenditure to keep down the amount of smoke, just the same as it costs money to provide sewage for our property, which the law prescribes, but these are expenses which the citizens have a right to ask and expect. Some will say, "we want smoke, it means industry." We say that a small amount of smoke is not inconsistent with industrial activity. After touching briefly on the contamination in various ways of the air we breathe, we are all agreed, I think, that vigilance should be the watch word.

1125 Main Street.

---

Aspiration of the Tympanic Cavity after Paracentesis; A Valuable Aid in the Treatment of Acute Otitis Media.—Percy Fridenberg, in his discussion of this subject describes a procedure which he has followed for about six years. Immediately after the paracentesis is made he presses against the external meatus, hermetically plugging the canal, a small glass bulb about five-eighths of an inch wide, shaped like an olive with a very blunt tip. Suction is then applied and gradually increased until there is a flow of fluid from the middle ear and the glass bulb becomes more or less filled. The effects of this aspiration by energetic suction are most gratifying. Pain is relieved. Drainage is far more free, several drams of fluid being often removed. The paracentesis opening does not have so great a tendency to become closed. The removal of inflammatory exudate from the middle ear and the dilution of the contents with blood serum also work against septic clot formation. In cases of very young children and infants, especially, when there is doubt whether the drum has been properly opened or not, the application of suction will immediately settle the question.—*Medical Record,* March 3, 1906.

